# Neonatal Acute Megakaryoblastic Leukemia Presenting with Leukemia Cutis and Multiple Intracranial Lesions Successfully Treated with Unrelated Cord Blood Transplantation

**DOI:** 10.1155/2015/610581

**Published:** 2015-07-01

**Authors:** Hiroshi Tsujimoto, Shinji Kounami, Yasuyuki Mitani, Takashi Watanabe, Katsunari Takifuji

**Affiliations:** ^1^Department of Pediatrics, Wakayama Medical University, 811-1 Kimiidera, Wakayama, Japan; ^2^Department of Second Surgery, Wakayama Medical University, 811-1 Kimiidera, Wakayama, Japan

## Abstract

Neonatal acute megakaryoblastic leukemia (AMKL) without Down syndrome (DS) is an extremely rare disorder. We report of a one-day-old male infant without DS who developed AMKL with leukemia cutis and right facial nerve palsy. Magnetic resonance imaging of the patient's brain revealed multiple intracranial tumors. A biopsy specimen of the skin lesion was suggestive of AMKL, but the bone marrow leukemic cells were less than 5% of the marrow nucleated cells. The skin and intracranial lesions had spontaneously regressed within one and a half months, but the patient's anemia and thrombocytopenia gradually worsened and the leukemic cells in the bone marrow gradually increased to more than 20% of the nucleated cells. In addition, multiple intracranial lesions reappeared at 72 days of life. We diagnosed the patient with AMKL, and chemotherapy followed by unrelated cord blood transplantation after a reduced-intensity conditioning regimen resulted in sustained complete remission. At present, the patient is well, and he has demonstrated normal development for five years.

## 1. Introduction

Neonatal or congenital leukemia is an extremely rare disorder that is diagnosed in the first 30 days after birth [1, 2]. The estimated incidence of neonatal leukemia ranges from one to five cases per million live births [3], and less than 1% of all cases of childhood leukemia are diagnosed in neonates. Neonatal leukemia is estimated to be the second most common malignancy in neonates and the leading cause of death from malignancy in this population [4]. Among the cases of neonatal leukemia, there have been a few reports of neonatal acute megakaryoblastic leukemia (AMKL) without Down syndrome (DS). Herein, we report a case of neonatal AMKL without DS, presenting with leukemia cutis and multiple intracranial lesions that was successfully treated with unrelated cord blood transplantation.

## 2. Case Presentation

**Figure 1:**
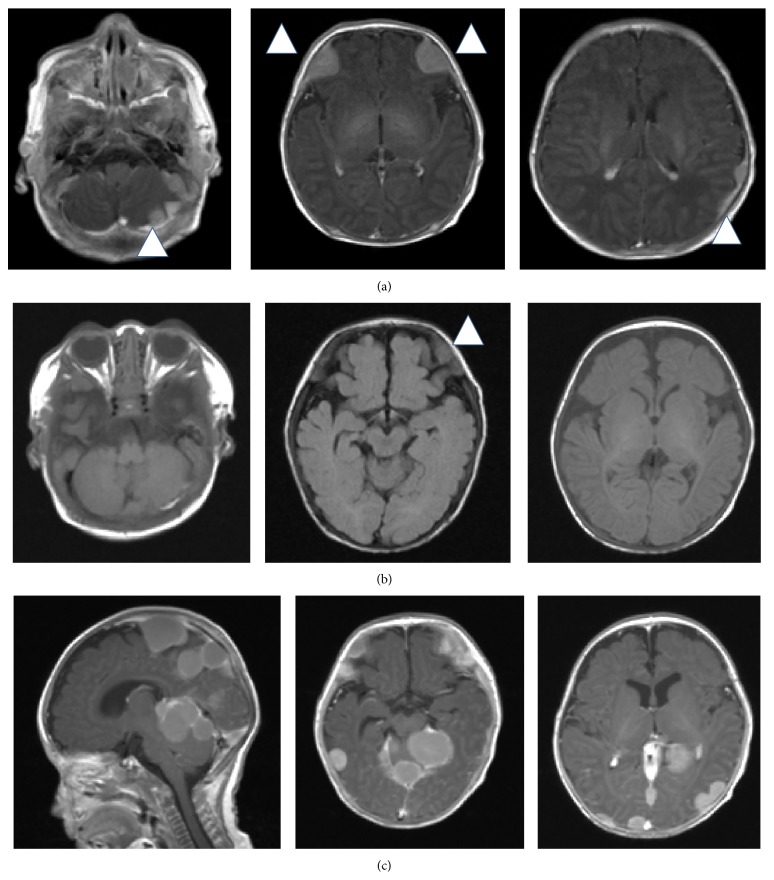
Magnetic resonance imaging studies of the brain: (a) a gadolinium-enhanced T1-weighted image at 7 days of age, (b) a T1-weighted image at 45 days of age, and (c) a gadolinium-enhanced T1-weighted image at 72 days of age.

**Figure 2:**
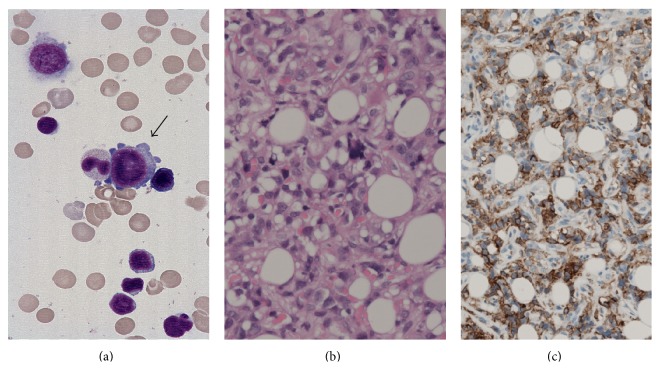
(a) Leukemic cells in the bone marrow aspirate (light-Giemsa staining). (b) A section of a biopsy specimen of the skin nodule (hematoxylin and eosin staining). (c) Immunostaining of the biopsy specimen of the skin nodule by using anti-CD42b antibodies.

A term male infant was born to a 28-year-old Japanese woman after an uncomplicated pregnancy. At birth, the infant was noticed to have skin nodules, petechiae, and right facial nerve palsy and he was referred to our hospital at one day of age. Examination revealed widespread dusky red papules and nodules (0.5 cm–1.5 cm in diameter) with ecchymosis predominantly on the patient's scalp, trunk, and distal extremities. In addition, he had right facial nerve palsy, but there was no hepatosplenomegaly or lymphadenopathy. There were no dysmorphic features, and the patient was later found to have a normal male karyotype by chromosome analysis on cultured peripheral blood lymphocytes and fibroblasts derived from buccal mucosa. Blood count on admission showed a white blood cell count of 1.63 × 10^9^/L, hemoglobin level of 12.6 g/dL, and a platelet count of 3.4 × 10^9^/L. A peripheral blood smear demonstrated 56% neutrophils, 4% monocytes, and 40% lymphocytes, with no blasts. The serum lactate dehydrogenase and C-reactive protein were 987 IU/L and 1.01 mg/dL, respectively. The results of the Coombs test, tests to rule out congenital infections,* Toxoplasma gondii*, rubella, cytomegalovirus, herpes simplex virus type 1, and parvovirus B19 infection, and blood cultures were negative. Magnetic resonance imaging of the brain showed multiple intracranial tumors (Figure 1). Cerebrospinal fluid was normal. A biopsy specimen of the skin nodule showed a diffuse infiltration of mononuclear cells with large oval nuclei. These cells stained positive for CD43, CD42b, and CD30 but negative for CD1a, CD68, CD34, CD56, CD99, and S-100 (Figure 2). Bone marrow aspiration revealed blasts with cytoplasmic blebs comprising 4.8% of the marrow nucleated cells (Figure 2). Cytochemistry results were negative for myeloperoxidase and periodic acid-Schiff staining. The blasts were positive for CD41 antigen on flow cytometric analysis. Cytogenetic studies of the bone marrow revealed hyperdiploid: 51, XY, +7, +8, +14, +19, +22 (10/20 cells). Serial chromosome analysis on bone marrow samples showed the same abnormality until the start of chemotherapy.* MLL* rearrangement and* RBM15-MKL1* fusion gene were negative.* GATA1* mutation analysis was not performed. We diagnosed the patient with AMKL presenting with leukemia cutis and multiple intracranial lesions, although the percentage of blasts was less than 20% of the nucleated cells.

The patient's skin lesions gradually disappeared within a month, and his facial nerve palsy recovered by 40 days in accordance with regression of the intracranial tumors (Figure 1). In contrast, anemia and thrombocytopenia gradually worsened, requiring repeated red blood cell and platelet transfusions. Bone marrow leukemic cells slowly increased to more than 20% of the marrow nucleated cells, and he had peripheral blasts. In addition, he again developed right facial nerve palsy and recurrence of intracranial tumors (Figure 1). Chemotherapy was initiated at 78 days, and the chemotherapy doses were reduced to two-thirds, with the doses calculated according to body weight instead of body surface area by considering 30 kg body weight to be equivalent to 1 m^2^ body surface area, pirarubicin 25 mg/m^2^ × 2 days, cytarabine 100 mg/m^2^ × 7 days, and etoposide 150 mg/m^2^ × 3 days [5]. The patient achieved complete remission after the first cycle of chemotherapy. After three cycles of chemotherapy, he underwent unrelated cord blood transplantation with a reduced-intensity conditioning regimen consisting of fludarabine (180 mg/m^2^) and melphalan (180 mg/m^2^). HLA 6/6-matched unrelated cord blood was administered because the patient had no other suitable donors. A combination of tacrolimus and short-term methotrexate was applied for GVHD prophylaxis. He maintained complete remission after the transplantation without any signs of graft-versus-host disease. His psychological development at 5 years was normal for his age (Tsumori-Inage Infant's Developmental Test), and his height and body weight were within normal limits, +0.15 standard deviation (SD) and +0.3SD, respectively.

## 3. Discussion

Neonatal leukemia is extremely rare, and most of the literature comprises case reports. Two publications reviewing 117 patients and 145 patients, respectively, reported that acute myeloid leukemia (AML) is more common than acute lymphocytic leukemia [6, 7]. Within neonatal AML, the most common French-American-British subtype is M5, followed by M4 and M7, which is seen less frequently. With the exception of transient abnormal myelopoiesis, found in neonates with DS, the occurrence of neonatal AMKL in infants without DS is very rare [6]. Neonatal AMKL is strongly associated with t(1;22)(p13;q13), a translocation found in approximately 70% of infant AMKL [8], forming the fusion gene* RBM15-MKL1*, and neonatal AMKL with t(1;22)(p13;q13) was seen in eight out of 13 non-DS neonatal AMKL patients reviewed by Isaacs Jr. [7]. The other five cases were associated with numerical abnormalities of chromosome 21, such as trisomy 21 mosaicism or isochromosome 21q [7]. The present case had numerical chromosome abnormalities without chromosome 21 abnormality that has not been reported in non-DS neonatal AMKL.

Neonatal leukemia has an unexplained natural tendency to undergo spontaneous remissions that last for months or sometimes years [9]. It is supposed that t(8;16)(p11;p13) translocation and a specific gene-expression profile have been associated with some of these spontaneous remissions [4]. Interestingly, in our case, the skin and intracranial lesions spontaneously regressed after birth, but the numbers of bone marrow blast cells gradually increased and the intracranial lesions reappeared, suggesting close monitoring is important.

The prognosis of neonatal leukemia is generally poor, and the survival rate of individuals with neonatal AML is estimated at approximately 25% [6, 7]. There are no reports of neonatal AML patients treated according to a uniform protocol, but considering that the outcome of infant AML has improved considerably with intensive chemotherapy [10, 11], the prognosis of neonatal AML might be better nowadays. There have been several reports concerning the outcome of childhood AMKL. Reinhardt et al. reported an improved outcome not different from that of non-AMKL patients with intensified induction chemotherapy [12]. On the other hand, it was reported that childhood AMKL patients without t(1;22)(p13;q13) had an inferior prognosis to that of non-AMKL patients [13] and that AMKL without t(1;22)(p13;q13) is categorized into the high-risk group of childhood AML [14]. Our patient underwent the less toxic chemotherapy regimen for AML with DS [5] and achieved complete remission without serious infectious complications. Although the role of hematopoietic stem cell transplantation in neonatal AML has not been confirmed [4], we diagnosed him as having high-risk AML and he underwent unrelated cord blood transplantation with a reduced-intensity conditioning regimen. Notably, the patient maintained complete remission and demonstrated normal physical and psychological development.

In conclusion, we have herein reported a rare case of neonatal AMKL without DS. To establish the appropriate treatment for neonatal leukemia, further studies may be needed.
